# Innovative work behavior in high-tech enterprises: Chain intermediary effect of psychological safety and knowledge sharing

**DOI:** 10.3389/fpsyg.2022.1017121

**Published:** 2022-10-24

**Authors:** Ziqing Xu, Sid Suntrayuth

**Affiliations:** International College, National Institute of Development Administration, Bangkok, Thailand

**Keywords:** innovative work behavior, psychological safety, knowledge sharing, organizational innovation climate, high-tech enterprises

## Abstract

This study aims to explore the relationship between organizational innovation climate (OIC) and innovative work behavior (IWB), using psychological safety (PS) and knowledge sharing (KS) as mediating variables. Based on the social cognitive theory (SCT), this study proposes a conceptual framework to explore innovative work behavior. The structural model of the extended SCT model was tested using sample data from 446 R&D staff of high-tech enterprises in China. SPSS 25.0 and AMOS 23.0 were used to test the hypothetical model. The results indicated that organizational innovation climate was positively correlated with psychological safety and innovative work behavior. Psychological safety was positively correlated with innovative work behavior. Knowledge sharing was significantly and positively correlated with innovative work behavior. Moreover, Psychological safety and knowledge sharing play a significant mediating role in the relationship between organizational innovation climate and innovative work behavior, and psychological safety further improves individual innovative work behavior by influencing knowledge sharing among research team members. At the end of the study, this study thoroughly discussed the conclusions, practical implications, limitations, and future research directions of the study.

## Introduction

Innovative work behavior in the High-tech industry is essential for organizational effectiveness and survival. R&D personnel is an important subject in high-tech enterprises, and their innovative work behavior directly affects the competitive advantage of enterprises (Liu et al., 2018; Mandych and Bykova, 2019). Out of this importance, innovative work behavior has been a heated topic in high-tech enterprises and technique development globally, as well as the rising academic interest (Bagheri, 2017). And no doubt exploring the predictors of innovative work behavior of R&D person shows great significance.

Organizational innovation climate and knowledge sharing influence innovative work behavior. Previous studies have discussed and debated it briefly. However, the relation to why R&D staff would like to have more innovative work behavior still needs more discussion. Especially in China, most staff prefer to work step by step. They tend to hide their different thinking from their leaders. At the same time, for innovative thinking, they tend to wait or observe for a while to judge whether this point is correct before sharing. In this organizational culture, the staff’s innovative behavior is inefficient. It also leads to a high psychological burden on staff’s innovative work behavior. This study is an effort to explain the mediating role of psychological safety and knowledge sharing between organizational innovation climate and innovative work behavior.

According to previous studies, the direct relationship between knowledge sharing and innovative work behavior has been investigated in previous studies. But the relationship between the organizational innovative climate, psychological safety, knowledge sharing, and innovative work behavior has not yet been explored. Particularly psychological safety and knowledge sharing as a mediating role need to be addressed and investigated by researchers. Due to the high power distance and collectivism in Eastern culture, many staffs tend to follow the arrangement of leaders and organizations in the process of work. When they have different views from others, they are reluctant to reveal true ideas. They worry that their behavior will not be approved by the leader and affect their career development, which reduces their desire to share and reduce their commitment to innovative work. So, this study is an effort to be helpful for the high-tech entrepreneurs of China to build an innovative climate to increase psychological safety and knowledge sharing to achieve innovative work behavior. This also will reveal how an organizational innovative climate affects staff’s psychological safety, and behavior, because an organization builds an innovative climate to help the R&D staff feel their innovative behavior is safe and encouraged.

Research on leadership relationships, knowledge sharing, innovative performance, and job anxiety of R&D staff in high-tech enterprises is currently receiving extensive attention. However, existing studies mostly focus on the causes of the workplace environment, leadership, and job anxiety problems, and verify the effectiveness of external factors in increasing staff’s innovative behavior (Sulistiyani and Rahardja, 2018; Zhang and Su, 2020; Yamin, 2022). Different from previous studies focusing on external factors, this study is the first study to focus on how an organizational innovative climate can improve staff’s psychological safety and knowledge sharing, and innovative work behavior. This study fills the current research gap by linking organizational stimulation with psychological activities and actual behavior, for the first time, quantitatively demonstrates the relationship between psychological safety, knowledge sharing, and innovative work behavior. Therefore, the objectives of this study are as follows: (1) To understand the psychological safety and knowledge sharing on how to influence innovative work behavior; (2) To explore the effect of organizational innovation climate on alleviating the innovative work behavior of R&D staff; (3) To report the existing organizational manage problems and make suggestions to the high-tech enterprises.

The rest of the study is structured as follows: Section Literature and review reviews the literature related to the theory of stimulus-organism-response and the hypotheses and the conceptual model of this study. Section Methodology describes the process and method of data collection. Section Results provides the results of data analysis and hypothesis testing. Section Discussion analyzes the results, and limitations and provides directions for future research. Section Conclusion summarizes the paper, and explain and discusses the findings.

## Literature review

### The social cognitive theory

**Figure 1:**
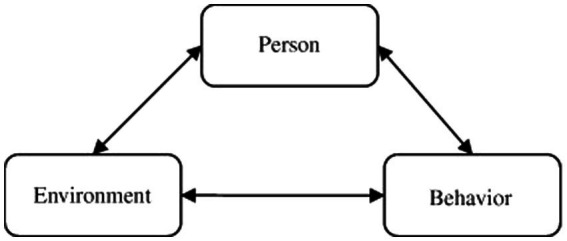
Social cognitive theory.

Social Cognitive Theory (SCT) has been widely used in verifying individual behavior, emphasizing that behavior, personal factor, and the environment are dynamic and interactive (Compeau and Higgins, 1995). Social cognitive theory (SCT) serves as the theoretical basis for the relationship between the three (see Figure 1), and personal factors, environmental influences, and behaviors influence each other in both directions (Wood and Bandura, 1989).

The purpose of this study was to empirically examine the innovative work behaviors of R&D personnel using Social Cognitive Theory (SCT). This study uses Social Cognitive Theory (SCT) as the basic theoretical framework to illustrate and explain how individual innovative work behaviors are formed. Innovative work behavior is a process driven by continuous learning. This study argues that social cognition can more powerfully explain the process of innovative work behavior. According to Bandura (1986), if individuals do not have confidence in their ability to share knowledge, they are less likely to do so, especially if knowledge sharing is voluntary. Therefore, this study that introduces the social cognitive theory takes organizational innovation climate as an environmental factor, and psychological safety as a personal factor, for an individual, psychological safety reflects the individual’s internal psychological state and self-perception.

Previous studies drawing on social cognitive theory (SCT) have overlooked the importance of the influence of organizational innovation climate, while research in the organizational innovation climate literature has paid less attention to the role of personal cognition, such as psychological safety. According to social cognition theory (SCT), why do individuals share their intangible property rights and good ideas with organizations and other people? Why do innovative work behaviors that may be risky? This problem should be solved from the perspective of individual cognition and organizational environment. However, social cognitive theory (SCT) is lacking in how organizational innovation climates affect individual innovative behavior through psychological safety. Therefore, Social Cognitive Theory (SCT) provides the basic framework for this concept, and this study also uses social cognitive theory to address the research question.

### Organizational innovation climate and innovative work behavior

In the 1930s, Lewin first proposed the concept of psychological climate, arguing that human behavior is inseparable from specific situations and living spaces, and people will have cognition of the social environment and then affect individual behavior. Litwin and Stringer (1968) developed the concept of organizational climate on this basis and believed that organizational climate is a collection of objective environmental factors that affect individual behavior and organizational performance. Based on the study of organizational climate, scholars put forward concepts such as organizational innovation climate and organizational psychological climate from the perspectives of innovation and psychology.

The conceptual definition of organizational innovation climate can be divided into subjective and objective perspectives. First, from an objective perspective, Kanter (1983) proposed that organizational innovation climate is a series of objective environmental factors that affect employee innovation activities. Another, subjective point of view. For Amabile et al. (1996), organizational innovation climate is the subjective perception of organizational members of the organizational innovation environment. Isaksen et al. (1999) further clarified that organizational innovation climate is the unified perception formed by individuals on the organization’s rules, regulations, and operational processes within the organization. At present, most scholars tend to define organizational innovation climate from a subjective perspective.

Innovative work behavior is the behavior of staff to introduce new and useful ideas in organizational activities and services. Amabile (1988) believes that innovative work behavior is a process in which people generate new ideas and realize new ideas, then use new ideas to solve problems. Lambriex-Schmitz et al. (2020) believed that innovative work behavior includes not only the generation and content of innovative ideas but also the promotion and implementation of innovative ideas; Kleysen and Street (2001) also believed that employee innovative work behavior is the purposeful introduction and apply new ideas to solve problems and promote them in the organization. Individual innovative behavior is defined as a complex set of actions consisting of three different activities in the workplace: generating, promoting, and realizing novel ideas (Scott and Bruce, 1994; Janssen, 2000).

Organizational innovation climate is an independent variable in research, and many scholars’ studies have shown that organizational innovation climate has a significant positive impact on innovative work behavior. Chih-Yang et al. (2011) confirmed through empirical research that the strength of staff’s cognition of their organizational innovation climate has a significant positive correlation with staff’s innovative behavior. Liu et al. (2019) also confirmed in the study that when the staff’s perceived organizational innovation climate is higher, they will have stronger work motivation and take the initiative to carry out more innovative behaviors. Therefore, this study proposes the following hypothesis:

*Hypothesis 1*: Organizational innovation climate can be positively associated with innovative work behavior.

### Psychological safety

The study of psychological safety originated from Schein and Bennis (1965) research on promoting organizational change. They argue that if an organization wants to implement a change strategy, it must create a work environment characterized by psychological safety to effect organizational change. Kahn (1990) believes that psychological safety is the belief of staff that when expressing and displaying their true self, they do not have to worry about the negative consequences of this behavior and affect their image or career. Considering that this study is mainly aimed at the innovative work behavior of R&D personnel in high-tech enterprises. This study defines psychological safety as the psychological perception that staff believes that their image or career will not be negatively evaluated when they engage in innovative behaviors, this is also a shared belief that staff perceives in their interpersonal interactions with colleagues and leaders. By summarizing the literature, this study for the first time incorporates the variable of psychological safety into the relationship between organizational innovation climate and innovative work behavior. Previous studies, more focused on the impact of leadership support (Javed et al., 2019); organizational climate (Andersson et al., 2020), staff voice (Miao et al., 2020), trust, and friendship (Basit, 2017) on psychological safety.

The organizational innovation climate encourages staff to fully display themselves, freely display their potential, and encourage and support staff to engage in innovative work. It can be said that organizations that support innovation develop and maintain a climate where members feel secure and free to experiment with new ideas, and where diversity of thought and opinion is valued (Shanker et al., 2017; Sofwan et al., 2021). Therefore, organizational innovation climate is important in forming personal psychological safety. This is mainly because the innovation climate encourages staff to propose new ideas, actively think about new problems, take meaningful risk-taking behaviors, and make mistakes or mistakes in innovation activities (Xu et al., 2022). If innovation fails, staff will not be punished or negatively pressured. This study proposes the following hypothesis:

*Hypothesis 2*: Psychological safety mediates the relationship between organizational innovation climate and innovative work behavior.

### Knowledge sharing

Knowledge sharing is an interaction between knowledge owners and knowledge demanders, and knowledge is passed between the owners and the demanders (Razmerita et al., 2016). Knowledge demanders enrich their knowledge reserves by accepting knowledge and transforming personal knowledge into organizational knowledge to maximize the value of knowledge (Radaelli et al., 2014; Abualoush et al., 2018; Ahmed et al., 2018). Organizational innovation climate is members’ perception that the organization encourages innovation. Szulanski points out that the more innovative the environment, the more willing staff are to interact with others and share knowledge. Anderson believes that organizational innovation climate can have an important impact on staff’s knowledge-sharing behavior. Knowledge sharing promotes communication and is a process of brainstorming, which in turn generates new knowledge and achieves the result that knowledge promotes innovation. Wei et al. (2020) believes that knowledge sharing can promote the exchange and communication of knowledge in the entire organization, and members learn from each other in communication and stimulate the generation of innovative ideas.

For the R&D personnel of high-tech enterprises, a good innovative organizational climate encourages staff to learn new things. Knowledge sharing can promote mutual help among staff and discuss technical difficulties encountered. Through brainstorming, new ideas and knowledge can be acquired faster and more efficiently, and ultimately promote the generation of innovative work behaviors. Therefore, this study proposes the following hypothesis:

*Hypothesis 3*: knowledge sharing mediates the relationship between organizational innovation climate and innovative work behavior.

### The chain intermediary effects

Psychological safety and knowledge sharing are two complex variables, and existing studies have found that they are affected by multiple factors, thus constituting a multi-level mediating variable. Andersson et al. (2020) believe that the psychological safety of team members plays an important role in the learning behavior of team members. Given that the reason why staffs are unwilling to share knowledge is their feelings of insecurity, past studies have examined this factor and consistently shown that psychological safety has a positive influence on employee knowledge sharing (Tang et al., 2015; Jiang et al., 2019; Men et al., 2020).

Although many studies have confirmed the respective influences of psychological safety and knowledge sharing on innovative work behavior, the internal relationship between psychological safety and knowledge sharing has not been discussed in depth in the model of organizational innovation climate affecting innovative work behavior. Therefore, this study will focus on discussing the influence paths and effects of psychological safety and knowledge sharing.

*Hypothesis 4*: Organizational innovation climate can indirectly predict the innovative work behavior through the chain mediating effect of psychological safety and knowledge sharing.

### Research framework

**Figure 2:**
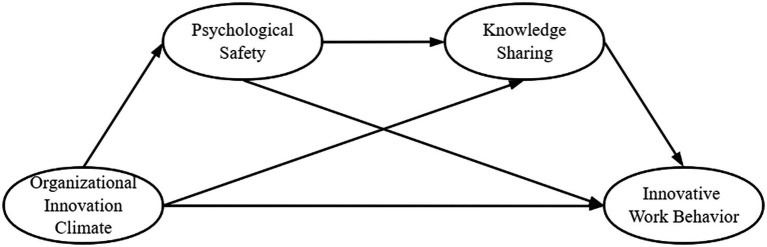
Conceptual framework.

This study constructs a chain mediating effect model between organizational innovation climate and innovative work behavior (see Figure 2), in which the independent variable is organizational innovation climate, the dependent variable is innovative work behavior, and the mediating variables are psychological safety and knowledge sharing.

How organizational innovation climate affects the innovative work behavior are as follows.: (1) Organizational innovation climate directly affects innovative work behavior. (2) Psychological safety and knowledge sharing play a mediating role between organizational innovation climate and innovative work behavior, respectively. (3) The organizational innovation climate affects knowledge sharing through psychological safety, affecting innovative work behavior.

## Methodology

### Sampling

This study used cluster sampling. The researchers first contacted the high-tech enterprise leaders of the Science and Technology Agency of Guangdong Province to obtain a list of high-tech enterprises in Guangdong Province. The researchers contacted 65 leaders of high-tech enterprises who were willing to participate in the survey by telephone. And through them, R&D staff who are willing to participate in this investigation were contacted. The researchers briefed all respondents about the purpose of the survey and informed them that the survey was anonymized and that all data was used for academic research only. In addition, all R&D staff who participated in the survey received a coffee voucher as a thank you. From June 2021 to January 2022, 550 questionnaires were distributed to the R&D staff of 65 high-tech enterprises in Guangdong Province. After excluding invalid questionnaires, a total of 446 valid questionnaires were recovered, with a recovery rate of 81.09%.

The background of the respondents was shown in Table 1. Among the participants, 255 were Male (57.2%) and 191 were female (42.8%). The mean age of respondents is 32 years old. Regarding work experience, 47.1% of the respondents have worked for “3 to 4 years.” Regarding education, most respondents are “masters,” accounting for 66.6%. Regarding job positions, 49.3% of the respondents chose “senior staff,” and 35.0% of the sample chose junior managers. The demographic information of this survey is close to the data published in the “China Statistical Yearbook 2021,” and the sample is representative.

**Table 1:** Participant profile (*N* = 446).

Measure	Items	Frequency(*n*)	Percentage(%)
Age	AVE = 32.425	446	–
Gender	Male	255	57.2
	Female	191	42.8
Tenure	Below 1 year	89	20.0
	1–2 years	45	10.1
	3–4 years	210	47.1
	4 years	49	11.0
	More than 4 years	53	11.9
Education	Below Bachelor	16	3.6
	Bachelor	114	25.6
	Master	297	66.6
	Doctor	19	4.3
Job position	Junior staff	61	13.7
	Senior staff	213	49.3
	Junior manager	139	35.0
	Middle manager	24	5.4
	Top manager	9	2.0

### Measures

The questionnaire consists of two parts, part 1 asked for information about the demographics of respondents. The second part was presented in Appendix A. To measure the effectiveness of organizational innovation climate(OIC), the study used a scale Zheng et al.’s (2009) developed, the questionnaire investigate from seven perspectives, such as incentive mechanism, team collaboration, leadership example, team cooperation, superior support, resource guarantee, organizational encouragement, and autonomous work. The measure of psychological safety (PS) was obtained from Detert and Burris (2007). Further, knowledge sharing (KS) was measured using the five-item scale Lu et al. (2006) developed, and innovative work behavior was measured using a scale Janssen (2000) developed. The four scales were measured using a five-point Likert scale (i.e., 1 = strongly disagree, 5 = strongly agree, or 1 = never, 5 = always).

To adapt to the research field and the specific cultural background, the researchers made certain adjustments to the scales’ items. To ensure the adjusted test scales’ reliability, a pilot test was conducted with R&D staff at High-Tech enterprises in Shenzhen City. The researchers distributed 50 questionnaires using the convenience sampling method and recovered 40 valid questionnaires. The results showed that Cronbach’s alpha coefficients were all greater than 0.8, indicating that the scales had good internal consistency.

### Data analysis

This study used IBM SPSS 25.0 for the preliminary data processing, descriptive statistics, and the reliability and correlation analyses among the variables. The AMOS 23.0 was used to analysis the chain mediation effect model, and the Bootstrap method was used to analyze the mediating effect of psychological safety and knowledge sharing in the relationship between organizational innovation climate and innovative work behavior.

## Results

### Measurement model

Reliability analysis included tests for Cronbach’s alpha coefficient and the composite reliability (CR) coefficient for the latent variables (Fornell and Larcker, 1981). As shown in Table 2, the variables had Cronbach’s alpha coefficients in the range of 0.862–0.919, well above the recommended value of 0.7. Convergent validity tests included factor loadings and extracted mean–variance (AVE). The AVE value of each construct in this study was, respectively, 0.556, 0.587, 0.616, and 0.556, which were all above 0.5. Therefore, all variables had high convergence validity. In addition, The measurement of discriminant validity requires verifying the relationship between the correlation coefficient of each latent variable and the square root of AVE. The square root value of AVE for all variables was more significant than the correlation coefficient between variables. The results are shown in Table 3; all correlation coefficients were less than the square root of AVE. Therefore, the variables demonstrated good discriminant validity.

**Table 2:** Reliability and validity tests.

Items	Loadings	Cα	AVE	CR
Organizational innovation climate		0.862	0.556	0.745
OIC1	0.746			
OIC2	0.648			
OIC3	0.706			
OIC4	0.710			
OIC5	0.618			
OIC6	0.776			
OIC7	0.736			
Psychological safety		0.919	0.587	0.766
PS1	0.818			
PS2	0.730			
PS3	0.753			
Knowledge sharing		0.895	0.616	0.784
KS1	0.770			
KS2	0.840			
KS3	0.795			
KS4	0.711			
KS5	0.691			
KS6:	0.714			
Innovative work behavior		0.901	0.556	0.745
IWB1	0.731			
IWB2	0.688			
IWB3	0.690			

All standardized loadings are significant at the 0.001 level.

**Table 3:** Discriminant validity among variables.

Variable	OIC	PS	KS	IWB
OIC	(0.745)	–	–	–
PS	0.418^**^	(0.766)	–	–
KS	0.391^**^	0.486^**^	(0.784)	–
IWB	0.434^**^	0.482^**^	0.559^**^	(0.745)

** *p* < 0.01.

*N* = 446. OIC, Organizational innovation climate; PS, Psychological safety; IM, Intrinsic motivation; KS, Knowledge sharing; IWB, Innovative work behavior.

### Structural path model

**Figure 3:**
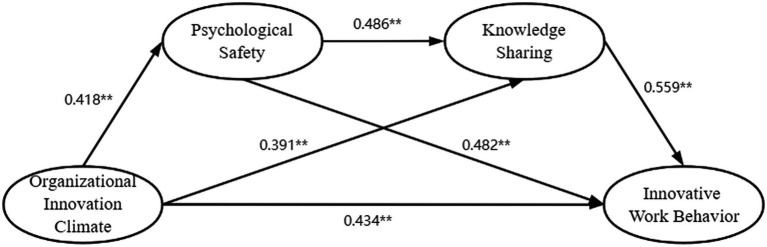
The chain mediation model. The chain mediation model shows the effects of organizational innovation climate, psychological safety, and knowledge sharing on innovative work behavior. *N* = 446. The effect of organizational innovation climate is shown in parentheses. In the PROCESS program of SPSS, the regression coefficient was obtained. **p < 0.01.

Since neither the error term nor the residual term of the structural model has negative values, it shows that the whole model does not violate the basic fitness test criterion. With reference to the suggested value of Hair et al. (2010) the structural model showed a good fit with the data (χ^2^/df = 1.265, GFI = 0.945, NFI = 0.940, CFI = 0.987, TLI = 0.985, RMSEA = 0.024). Significant and positive correlations were found between the independent variables, the mediators, and the dependent variables, which provides preliminary support for the verification of the research hypotheses. The structural path model results are presented in Figure 3; the effect of organizational innovation climate on innovative work behavior was statistically significant (β = 0.434, *p* < 0.001), supporting H1 (Table 4).

**Table 4:** The structural model.

Fitting index	χ^2^/df	GFI	AGFI	NFI	TLI	CFI	RMSEA
Reference standard value	<3	>0.8	>0.8	>0.8	>0.9	>0.9	<0.08
Statistical value of this study	1.265	0.945	0.932	0.940	0.985	0.987	0.024
Conclusion	Support	Support	Support	Support	Support	Support	Support

### Chain mediation model analysis

This study used the bootstrapping approach to verify the mediating effects. The Bootstrap test program (PROCESS plug-in) in SPSS25.0 developed by Hayes (2012) was used to test 74 theoretical (research) models. Model 4 and Model 6 in SPSS 25.0 was used to analyze the mediation effect. Set “Bootstrap sample” to 5,000, indicating the sampling times; The confidence interval is generally 95%, and the higher the confidence interval is, the higher the confidence degree is. Among them, “Bia Corrected” is selected as the sampling method. Figure 3 is a chain mediating model between organizational innovation climate and innovative work behavior. Table 5 and Figure 3 show that psychological safety and knowledge sharing play a significant mediating role between psychological empowerment and retention intention.

**Table 5:** Mediation effect analysis.

Path	Effect	Boot SE	Boot LLCI	Boot ULCI	z	*P*
OIC → PS → IWB	0.087	0.019	0.051	0.126	4.597	0.000(^***^)
OIC → KS → IWB	0.144	0.008	0.032	0.064	5.517	0.000(^***^)
OIC → PS → KS → IWB	0.163	0.012	0.040	0.086	5.408	0.000(^***^)

Standardized estimation of 5,000 bootstrap samples.

*** *P* < 0.001.

The conceptual model suggests that organizational innovation climate positive impacts on innovative work behavior through two mediators (i.e., psychological safety and knowledge sharing). The results of 5,000 bootstrap samples, with a 95% confidence interval, are presented in Table 5; all *Z*-values were greater than 1.96, and there was no zero value in the 95% confidence interval. Moreover, it showed that significant mediation occurred between organizational innovation climate (OIC) and innovative work behavior(IWB) through psychological safety (PS) (standardized indirect effect = 0.087, *p* < 0.001), which provides support to hypotheses 2. It also showed that significant mediation occurred between organizational innovation climate (OIC) and innovative work behavior(IWB) through knowledge sharing(KS; standardized indirect effect = 0.144, *p* < 0.001), which provides support to hypotheses 3. Meanwhile, the mediating path of organizational innovation climate (OIC) and innovative work behavior(IWB) through two mediators (i.e., psychological safety and knowledge sharing). The results shows hypotheses 4 is supported(standardized indirect effect = 0.163, *p* < 0.001). The findings mean that R&D staff who have a high perception of the organizational innovation climate and a positive psychological safety can engage in knowledge sharing and innovative work behavior.

## Discussion

### Contributions

First, the positive impact of organizational innovation climate on innovative work behavior is explained from the perspective of social cognitive theory (SCT). Most of the previous studies have ignored the role of organizational environment on innovative behavior, focusing more on personal factors (i.e.,: leadership style, work engagement). On the one hand, this study starts with exploring the antecedents that affect innovative work behavior and uses social cognitive theory (SCT) to deeply analyze how organizational innovation climate as an environmental factor affects innovative work behavior, which is conducive to improving researchers’ attention and understanding of organizational innovation climate. On the other hand, this study also explores the internal factors that affect innovative work behavior. This study also supports previous studies on the positive impact of psychological safety on knowledge-sharing behavior (Kakar, 2018; Wang et al., 2018; Gerpott et al., 2021) and the impact of knowledge sharing on innovative work behavior (Akram et al., 2020; Asurakkody and Kim, 2020) enriching related research on innovative work behavior.

Second, it deeply analyzes the influence process of organizational innovation climate on employees’ innovative work behavior. Previous studies have mostly focused on a single perspective. On the one hand, this study confirms the mediating role of psychological safety and knowledge sharing between organizational innovation climate and innovative work behavior by giving social cognitive theory (SCT), psychological safety is used to represent individual cognition, and the overall conduction path between the research variables is formed through the logical process of environment-cognition-behavior. Therefore, this study not only expands the study of Azeem et al. (2021) on the mediating role of knowledge sharing between organizational climate and innovative behavior but also verifies the influence of psychological safety mediating effect on innovative work behavior (Javed et al., 2019). Moreover, this study supports and further expands the social cognitive theory (SCT) model, that is, organizational innovation climate, as an environmental factor, can positively predict psychological safety, and psychological safety can positively predict knowledge sharing, which has a positive impact on innovative work behavior.

### Practical implications

This study contributes two practical insights. Firstly, organizational innovation climate has a positive effect on innovation work behavior. Therefore, enterprises should strengthen the construction of an internal organizational innovation climate, and enhance resource support, leadership support, and team communication. Because innovation needs a lot of time, energy, and resources, it is a risky activity with a high possibility of failure. Enterprises should provide good funds, technology, and equipment foundation for employees’ innovation, and protect the interests of employees’ innovative work behavior in terms of rules and regulations so that organizational support becomes a solid backing for employees’ innovative behavior. In addition, managers should build an innovation culture that encourages innovation and tolerates failure. Managers should provide positive and effective communication and feedback channels for employees, help them solve the difficulties encountered, and reward and commend employees’ innovative behaviors and achievements in time.

Second, from the research results, it can be found that staff’s psychological safety and knowledge sharing have a very important mediating effect that affects the individual’s innovative behavior. Therefore, enterprises need to focus on staff’s psychological safety and knowledge sharing in innovation management. In terms of knowledge sharing, Organizations can build a knowledge-sharing platform through technical and tool support to further promote the sharing of resources among scientific researchers, especially the mutual exchange of new ideas and methods. Unlike the communication characteristics of explicit knowledge, sharing tacit knowledge is more conducive to scientific researchers to complete team innovation tasks and improve the effectiveness of innovation practice. Based on the feedback from the returned questionnaires, the researchers found that under the influence of East Asian culture (e.g., high power distance), staff dare not express their true thoughts with their leaders when their thoughts are inconsistent. This is because most staff are worried that they might get negation and dislike their leaders when expressing different views, and they might even negatively influence their work. To improve the psychological safety of staff in East Asian countries, leaders should first let staff know that it is safe to express real ideas in the company and that they will not be punished if they raise the company’s problems and even get rewards if the ideas are adopted. These methods can effectively improve staff’s psychological safety in the work of innovative behavior.

### Limitations and future research

The main limitation of this study is that the survey samples are concentrated in the R&D staff of high-tech enterprises in Guangdong province, China, and cross-sectional data is used. The survey samples are not continuously tracked in stages, and their dynamic development process cannot be well revealed.

In future research, on the one hand, the scope of research can be expanded, and the research can be divided into different categories; on the other hand, comparative research can be carried out through continuous tracking of longitudinal data. At the same time, other mediating or moderating variables can be identified, the relationship between these variables can be deeply explored, and the influence of the relationship model between organizational innovation climate and innovative work behavior in different periods and different regions can be studied.

## Conclusion

This study offers some major contributions to the existing literature by testing the concepts developed in a non-western culture. Innovation is the core of high-tech enterprises, but in Eastern culture research, there are relatively few studies on organizational innovation climate and employee innovative work behavior. At the same time, the effects of variables such as psychological safety and knowledge sharing in organizational innovation climate and innovative work behavior are not completely clear. Therefore, This is the first time that research has demonstrated organizational innovation climate positively influences innovative work behavior through psychological safety and knowledge sharing.

Moreover, this study based on social cognitive theory (SCT) constructs a chain mediation model that organizational innovation climate effect innovative work behavior, and psychological safety and knowledge sharing as the mediating variables. It studies the psychological safety and knowledge sharing of R&D staff motivated by the organizational innovation climate and then affects their innovative work behavior. This study was based on the social cognitive theory (SCT), and the results of the current study prove that the social cognitive theory (SCT) supports the above-mentioned relationship.

The main conclusions are as follows:(1) There is a direct positive correlation between organizational innovation climate and innovative work behavior; (2) Psychological safety and knowledge sharing play a significance mediating role between organizational innovation climate and innovative work behavior; (3) For R&D staff, the better the organizational innovation climate, the stronger the sense of psychological safety for innovation, and the more conducive to increasing the knowledge sharing willingness of R&D staff with other team members, thereby affecting their innovative work behavior; (4) The organizational innovation climate affects knowledge sharing through psychological safety, which in turn affects innovative work behavior. By discussing the impact of organizational innovation climate on innovative work behavior, this study reveals managers should pay more attention to psychological safety and knowledge sharing in high-tech enterprises.

## Data availability statement

The original contributions presented in the study are included in the article/supplementary material, further inquiries can be directed to the corresponding author.

## Author contributions

Conceptualization, methodology, formal analysis, data curation, funding acquisition, writing—original draft preparation, ZX, methodology, writing—review and editing, supervision, SS. All authors have read and agreed to the published version of the manuscript.

## Funding

This study was supported by grants from the National Institute of Development Administration, Thailand.

## Conflict of interest

The authors declare that the research was conducted in the absence of any commercial or financial relationships that could be construed as a potential conflict of interest.

## Publisher’s note

All claims expressed in this article are solely those of the authors and do not necessarily represent those of their affiliated organizations, or those of the publisher, the editors and the reviewers. Any product that may be evaluated in this article, or claim that may be made by its manufacturer, is not guaranteed or endorsed by the publisher.

## Appendix

**Appendix A Research Instrument**

**Table:**

**Organizational innovation climate**	
OIC1	The company's reward system makes everyone full of enthusiasm for innovation.
OIC2	My leaders often work creatively.
OIC3	Colleagues often communicate and discuss issues at work.
OIC4	My ideas at work can be supported by leaders.
OIC5	My innovative ideas can be supported by the corresponding resources.
OIC6	The company encourages everyone to use new methods to solve problems.
OIC7	I can decide for myself how to implement the work plan.
**Psychological safety**	
PS1	It is safe to tell the truth in a company.
PS2	Point out that problems (including serious problems) in the company will not be punished.
PS3	Making mistakes can be tolerated and accepted by the company.
**Knowledge sharing**	
KS1	In my daily work, I take the initiative to impart business knowledge to colleagues.
KS2	I share useful work experience and ideas with everyone.
KS3	After learning new knowledge useful for work, I share it so that more people can learn it.
KS4	At work, I take out my knowledge to share with more people.
KS5	I actively use the company's existing information technology to share my knowledge.
KS6	As long as other colleagues need it, I always say all my know and say it without reserve.
**Innovative work behavior**	
IWB1	I often look for new working methods, techniques, or tools.
IWB2	I often encourage colleagues to enthusiastically pursue innovative ideas.
IWB3	I often turn innovative ideas into something practical.
